# Factors affecting the association between overall survival and progression-free survival in clinical trials of first-line treatment for patients with advanced non-small cell lung cancer

**DOI:** 10.1007/s00432-014-1617-3

**Published:** 2014-02-22

**Authors:** Masayuki Aboshi, Masayuki Kaneko, Mamoru Narukawa

**Affiliations:** Department of Clinical Medicine (Pharmaceutical Medicine), Kitasato University Graduate School of Pharmaceutical Sciences, Shirokane 5-9-1, Minato-ku, Tokyo, 108-8641 Japan

**Keywords:** Meta-analysis, Overall survival, Progression-free survival, Post-progression survival, Non-small cell lung cancer

## Abstract

**Purpose:**

New treatment strategies, particularly the introduction of molecular-targeted agents and appropriate patient selection based on histology and/or genotyping, have progressed markedly in recent years, and the overall survival (OS) in advanced non-small cell lung cancer (NSCLC) patients has improved. The aim of the study was to identify factors affecting longer OS than that estimated from progression-free survival (PFS) in first-line treatment for advanced NSCLC.

**Methods:**

Sixty-five controlled trials for first-line treatment of advanced NSCLC were extracted for the study. Factors influencing higher than predicted OS were examined by logistic regression analysis between the OS-extended group and the OS-association group.

**Results:**

PFS was moderately associated with OS. Twenty arms of 14 trials were categorized as an OS-extended group, in which the ratio of observed OS to estimated OS was found to be over 1.2. On multivariate logistic regression analysis, number of patients lower than 150, average age younger than 63 years, and percentage of squamous carcinoma <30 % were found to significantly affect this relationship.

**Conclusion:**

We identified number of patients and well-known prognostic factors including age and histological cancer type as factors influencing longer OS. These factors should be considered for patient eligibility, when PFS is used as a surrogate primary endpoint for OS in randomized clinical trials of first-line treatment for patients with advanced NSCLC.

**Electronic supplementary material:**

The online version of this article (doi:10.1007/s00432-014-1617-3) contains supplementary material, which is available to authorized users.

## Introduction

Lung cancer is the leading cause of cancer death worldwide, and most lung cancers are non-small cell lung cancer (NSCLC) (Jemal et al. 2010). More than half of all NSCLCs are diagnosed at an advanced stage; therefore, prognosis is often poor (National Cancer Institute 2013). In recent decades, platinum-based doublet chemotherapy has been used as first-line treatment for patients with advanced NSCLC (Hotta et al. 2004; Lilenbaum et al. 2005; D’Addario et al. 2005; Azzoli et al. 2009). However, such treatment is associated with only modest improvements in overall survival (OS) and quality of life (QOL) (Schiller et al. 2002; Rajeswaran et al. 2008). New treatment strategies, particularly the introduction of molecular-targeted agents and appropriate patient selection based on histology and/or genotyping, have resulted in marked progress in recent years, and OS in advanced NSCLC patients has improved (Ramalingam et al. 2011; Kobayashi and Hagiwara 2013; Kaneda et al. 2013). Although there are reports in the literature of OS of over 24 months (Arrieta et al. 2010; Maemondo et al. 2010; Niho et al. 2012), currently available treatments are not able to cure; attempts to find a curative treatment are ongoing (Black and Morris 2012; Berardi et al. 2013; Bayraktar and Rocha-Lima 2013).

OS is the gold standard endpoint for clinical trials assessing the efficacy of new drugs for the treatment of NSCLC (Food Drug Administration 2011). OS is defined as the time from random assignment of the first treatment to death. Measuring OS as an endpoint in clinical trials requires large number of patients and increasing longer follow-up, thus potentially increasing the cost of development and delaying time to approval. Additional issues with OS as an endpoint include the confounding impact of therapies given upon progression, and death not related to cancer (Garon 2012). Progression-free survival (PFS) has become an accepted alternate endpoint in assessing efficacy in advanced NSCLC (Food Drug Administration 2011; Garon 2012; Johnson et al. 2011). However, there are examples of improvement in PFS without an OS benefit (Lima et al. 2009), and an OS benefit without PFS improvement (Cheema and Burkes 2013). Recent well-developed multiple lines of therapies for advanced NSCLC patients after progression with first-line treatment have been increasing post-progression survival (PPS). Moderate to large improvement in PPS has resulted in reducing or losing an OS benefit in comparative clinical trials of first-line treatment, even though significant PFS benefit is elucidated (Lima et al. 2009). Therefore, PFS is probably the only rational endpoint for the current clinical trials, particularly in crossover design (Booth and Eisenhauer 2012; Mok 2011).

Hotta et al. (2011) and Hayashi et al. (2012) reported that PPS was highly associated with OS in first-line chemotherapy for advanced NSCLC, whereas the correlation between PFS and OS was moderate. This relationship was also found in second- and third-line chemotherapy for advanced NSCLC (Hayashi et al. 2013). PPS may be one of the factors reducing the OS benefit, but little is known regarding what factors other than PPS affect the association between OS and PFS. The aim of the present study was to identify factors affecting the association between OS and PFS, particularly those causing longer OS than that estimated based on PFS.

## Materials and methods

### Trial selection and database construction

Controlled trials for first-line treatment of advanced NSCLC published between January 1, 2003 and December 31, 2012 were identified through a systemic search of PubMed. Keywords used were “controlled clinical trial,” “first-line treatment” and “NSCLC.” The results were limited to articles published in English.

All retrieved abstracts were reviewed in accordance with pre-specified inclusion and exclusion criteria. Included studies were randomized phase II or III clinical trials of first-line therapy of advanced NSCLC that presented results for both OS and either PFS or time to progression (TTP). Studies were excluded if the treatment was adjuvant, maintenance or second-line therapy. Studies that investigated only immunotherapy regimens and those that were designed to assess combined modality treatment including radiation therapy and surgery were also excluded. To avoid bias, two observers (MA and MK) independently abstracted the data from the articles.

### Data abstraction

Abstract data included publication year and reference, patient characteristics (age, gender, race, Eastern Cooperative Oncology Group Performance Status (PS), disease stage, histological type of NSCLC, smoking history, and genotype), treatment information (chemotherapy and/or molecular-targeted regimen), trial characteristics (study phase, study period, region (with/without Asian countries), number of countries, number of sites, and number of patients), and efficacy information (median months, PFS, TTP, OS). Percentages of males, PS 1, disease stage IV, and squamous cell carcinoma were used as variables for gender, PS, disease stage, and histological type of NSCLC, respectively. OS and either PFS or TTP were determined for all the treatment arms using published data or survival curves. PFS and TTP were collectively referred to as PFS in order to increase the sample size as done previously in recent reports (26,27). PPS was defined as OS minus PFS for each trial.

### Data analysis

To assess the correlation between OS and either PFS or PPS, we used Spearman’s rank correlation coefficient (*r*). We conducted a simple linear regression analysis to obtain a regression line between PFS and OS and calculated the estimated OS from the PFS based on the regression equation. According to the ratio of observed OS/estimated OS, the treatment arms were classified into three groups: <0.8 (OS-reduced group), 0.8–1.2 (OS-association group), and >1.2 (OS-extended group). Factors influencing higher and lower than predicted OS were initially examined by univariate logistic regression analysis using a fixed-effect model between the OS-extended group and the OS-association group as well as the OS-reduced group and the OS-association group. After identifying potential influencing factors, multivariate logistic regression analysis was conducted in a stepwise fashion to further investigate factors that contribute to OS extension. A *p* < 0.05 was considered statistically significant throughout the analyses except where otherwise noted. The analyses were conducted using StatsDirect software (ver. 2.7.9; StatsDirect Ltd. UK).

## Results

### Characteristics of the trials

**Fig. 1:**
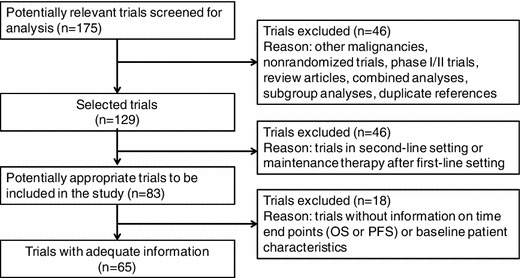
Flow chart showing the progress of trials through the review

A total 175 potentially relevant trials were identified. Initially, 46 trials were excluded for at least one of the following reasons: other malignancies, non-randomized, phase I/II, review articles, combination analyses, subgroup analyses, and duplicate references. A further, 46 trials were excluded because the trials were in a second-line setting or involved maintenance therapy after first-line treatment. Finally, after excluding trials without information on the necessary endpoints (OS, PFS or TTP) or patient baseline characteristics, 65 trials were considered to be highly relevant for the present study. The selection process for the randomized controlled trials is shown in Fig. 1.

The main characteristics of the 65 trials are listed in Table 1. A total of 140 treatment arms and 23,337 patients with advanced NSCLC were included. The median of the number of patients per trial was 70.5 (range 20–863) with most trials having a high proportion of males (71.0 %: range 20.5–95.7 %). The average median age was 61.1 years (range 56–78). Among 140 treatment arms, there were 86 chemotherapy arms, 7 molecular-targeted therapy arms, and 47 combination therapy arms. Eighty three arms were from phase II trials and 57 from III trials. Trials were classified into two groups by the year of start of the trial, considering the timing of the introduction of molecular-targeted agents: between 1998 and 2003 (59 arms), and between 2004 and 2008 (67 arms). The median OS was 9.9 months (range 3.5–30.5), and median PFS was 5.0 months (range 1.7–10.8) for all arms. Information of the number of participating counties and the race of trial subjects was very limited. There was limited data on genotyping of NSCLC and treatment after progression.

**Table 1:** Characteristics of 140 treatment arms in the 65 trials

Variables	Overalls
Total number of trials	65
Total number of arms	140
Total number of patients	23,337
Treatment	
Chemotherapy	86 (61.4 %)
Molecular target therapy	7 (5.0 %)
Combination therapy	47 (33.6 %)
Phase	
II	83 (59.3 %)
III	57 (40.7 %)
Study period	
1998–2003	59 (42.1 %)
2004–2008	67 (47.9 %)
Unknown	14 (10.0 %)
Region	
Including Asian countries	20 (14.3 %)
Without Asian countries	55 (39.3 %)
Unknown	65 (46.4 %)
Number of sites (*n* = 65)	55.0 (3–200)
Average of age (*n* = 136)	62.8 (56.0–78.0)
Percentage of male patients (*n* = 140)	69.1 (20.5–95.7)
Percentage of patients with PS 1 (*n* = 113)	56.0 (0–83.0)
Percentage of patients with stage IV (*n* = 128)	81.2 (44.0–98.0)
Percentage of patients with squamous cell carcinoma (*n* = 128)	26.4 (0–64.0)
Percentage of smoker (*n* = 55)	70.7 (6.3–100)
OS (months) (*n* = 140)	10.6 (3.5–30.5)
PFS (months) (*n* = 140)	5.0 (1.7–10.8)

### Relation between OS and either PFS or PPS

**Fig. 2:**
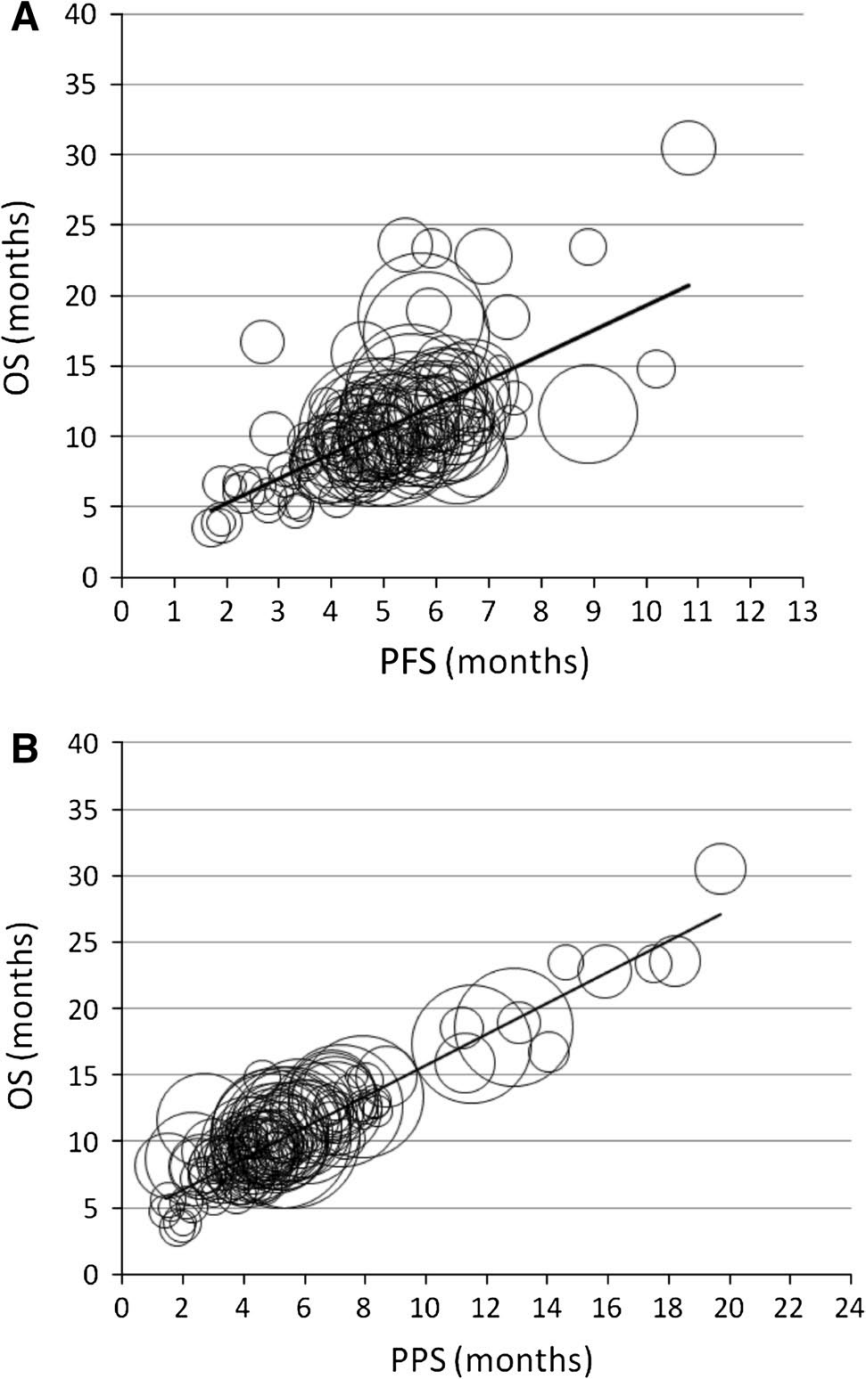
Correlation between Overall Survival (OS) and either (**a**) Progression-Free Survival (PFS) or (**b**) Post-Progression Survival (PPS) for 140 arms of 65 clinical trials for first-line treatment for patients with advanced NSCLC. The coefficients of correlation (*r*) between OS either PFS or PPS were 0.662 and 0.935, respectively. The size of each *circle* is proportion to the number of patients in the corresponding arm

The relation between OS and either PFS or PPS for the 140 arms is shown in Fig. 2. PPS was strongly associated with OS (Spearman’s *r* = 0.841, *p* < 0.0001), whereas PFS was more moderately associated with OS (*r* = 0.689, *p* < 0.0001). The regression line between OS and PFS was:

$${\text{OS }} = { 1}. 80 1 { } + { 1}. 7 4 9 { } \times {\text{ PFS}}\quad \left( {r^{ 2} = \, 0. 4 3 9} \right)$$

### Characteristics of the OS-extended group

Based on the ratio of the observed OS to the estimated OS, we classified the treatment arms into three groups: OS-reduced group (ratio: <0.8), OS-association group (ratio: 0.8–1.2), and OS-extended group (ratio: >1.2). Characteristics of the three groups are summarized in Table 2. There were 20 arms (14.3 %) from 14 trials in the OS-extended group (Arrieta et al. 2010; Maemondo et al. 2010; Niho et al. 2012; Hirsch et al. 2011; Park et al. 2007; Mok et al. 2009a, b; Heymach et al. 2008; Grossi et al. 2012; Gridelli et al. 2007; Gebbia et al. 2010; Chen et al. 2007; Lilenbaum et al. 2008; Ramlau et al. 2008; Table 3). The range of the observed OS/estimated OS ratio was 1.22–2.75. In the OS-association group, there were 94 arms of 53 trials, and in the OS-reduced group, there were 26 arms of 18 trials. After excluding the arms from the OS-extended group, the correlation between OS and PFS improved (Spearman’s *r* = 0.789, Supplement Figure 1).

**Table 2:** Characteristics of each category classified by the ratio of observed OS/estimated OS

Ratio of OS observed/estimated	OS			*p* value^a^
	Reduced group	Association group	Extended group	
	<0.8	0.8–1.2	>1.2	
Total of trials	18	53	14	
Total of arms	26	94	20	
Total of patients	2,576	18,271	2,490	
Treatment				
Chemotherapy	15 (57.0 %)	60 (63.8 %)	11 (55.0 %)	0.459
Others^b^	11 (43.0 %)	34 (36.2 %)	9 (45.0 %)	
Phase				
II	18 (69.2 %)	48 (51.1 %)	17 (85.0 %)	0.005
III	8 (30.8 %)	46 (48.9 %)	3 (15.0 %)	
Study period				
1998–2003	17 (65.4 %)	33 (35.1 %)	6 (30.0 %)	0.459
2004–2008	9 (34.6 %)	48 (51.1 %)	13 (65.0 %)	
Unknown	0	13 (13.8 %)	1 (5.0 %)	
Region				
Including Asian countries	0 (0.0 %)	11 (11.7 %)	9 (45.0 %)	0.001
Without Asian countries	13 (50.0 %)	38 (40.4 %)	4 (20.0 %)	
Unknown	13 (50.0 %)	45 (47.9 %)	7 (35.0 %)	
Number of sites per trial	18.2	69.9	31.0	
<30	7	15	9	0.028
≧30	2	28	4	
Number of patients per arm	99.1	194.4	124.5	
<150	21	51	17	0.011
≧150	5	43	3	
Average of age (year)	65.1	62.9	59.7	
<63 year	14	55	16	0.014
≧63 year	10	39	2	
Percentage of male patients^c^	72.7 %	70.9 %	56.1 %	
<70 %	10	35	15	0.002
≧70 %	16	59	5	
Percentage of patients with PS 1^c^	53.2 %	56.9 %	55.0 %	
<60 %	14	40	8	0.442
≧60 %	3	37	11	
Percentage of patients with stage IV^c^	83.1 %	81.2 %	78.8 %	
<80 %	5	32	11	0.097
≧80 %	18	54	8	
Percentage of patients with squamous cell carcinoma^c^	28.8 %	28.4 %	12.6 %	
<30 %	13	46	16	0.002
≧30 %	12	40	1	
Percentage of smokers^c^	63.9 %	76.2 %	57.6 %	
<70 %	2	4	9	<0.001
≧70 %	3	33	4	
OS (months)	7.9	10.0	16.6	
PFS (months)	5.2	4.9	5.2	

^a^*p* value was obtained by chi-square test between the OS-association group and the OS-extended group (>1.2)

^b^Others consists of molecular-targeted agents and the combination of chemotherapy and molecular-targeted agents

^c^Percentage per arm

**Table 3:** Design and characteristics of trials of the OS-extended group

Source		Treatment	Phase	No. of sites	No. of patients	Age years	Male (%)	PS 1 (%)	Disease stage IV (%)	Squamous (%)	OS^a^	PFS^a^
Hirsch et al. (2011)	Chemo^b^ + TKI^c^	Carboplatin/Paclitaxel + Erlotinib	2	NA^d^	71	61	56	NA	NA	NA	16.7	2.7
Maemondo et al. (2010)	Chemo	Carboplatin/Paclitaxel	2	43	114	62.6	36	48.2	73.7	1.8	23.6	5.4
	TKI	Gefitinib	2	43	114	63.9	36.8	51.8	77.2	2.6	30.5	10.8
Niho et al. (2012)	Chemo	Carboplatin/Paclitaxel	2	19	59	60	64	51	71	0	23.4	5.9
	Chemo + MAB^e^	Bevacizumab + Carboplatin/Paclitaxel	2	19	121	61	64	49	69	0	22.8	6.9
Park et al. (2007)	Chemo	Cisplatin/Taxane or Gemcitabine	3	15	156	56	63.5	5.5	78.8	26.3	15.9	4.6
Mok et al. (2009a)	TKI	Gefitinib	3	87	609	57	20.5	64.2	75.4	0	18.6	5.7
	Chemo	Carboplatin/Paclitaxel	3	87	608	57	20.9	62.8	76.2	0	17.3	5.8
Mok et al. (2009b)	Chemo	Gemcitabine/Cisplatin	2	19	78	57	69	71	79	0	18.9	5.9
	Chemo + TKI	Gemcitabine/Cisplatin + Erlotinib	2	19	76	57.5	71	62	83	0	18.5	7.4
Heymach et al. (2008)	TKI	Vandetanib	2	NA	73	63	67	70	86	22	10.2	2.9
Grossi et al. (2012)	Chemo	Q3W^f^ Cisplatin/Docetaxel then Gemcitabine	2	15	41	60	73	37	85	15	12.3	3.9
Arrieta et al. (2010)	Chemo + Other MT^g^	Cisplatin/Paclitaxel + ATRA^h^	2	NA	52	59.5	51.9	80.8	75	19.2	23.5	8.9
Gridelli et al. (2007)	Chemo	Gemcitabine/Docetaxel	2	NA	39	56.5	46.1	76.9	79.4	20.5	12.8	4.5
Gebbia et al. (2010)	Chemo	Docetaxel/Cisplatin	2	NA	42	61	76	83	81	29	12.1	4.2
	Chemo	Vinorelbine/Cisplatin	2	NA	44	62	80	82	80	34	12.5	4.5
Chen et al. (2007)	Chemo	Docetaxel/Cisplatin	2	NA	46	60.2	56.5	71.7	80.4	26.1	13.0	4.7
Lilenbaum et al. (2008)	TKI	Erlotinib	2	14	52	NA	44	0	87	NA	6.6	1.9
	Chemo	Paclitaxel/Carboplatin	2	14	51	NA	55	0	86	NA	9.7	3.5
Ramlau et al. (2008)	Vaccine + Chemo	TG4010 + Cisplatin/Vinorelbine	2	9	44	58.5	70.5	77.3	75	18.2	12.7	4.8

^a^Median months

^b^*Chemo* chemotherapy

^c^*TKI* tyrosine kinase inhibitor

^d^*NA* not available

^e^*MAB* monoclonal antibody

^f^*Q3W* once every 3 weeks

^g^*MT* molecular-targeted treatment

^h^*ATRA* all *trans*-retinoic acid

Statistically significant differences on univariate logistic regression analysis between the OS-association group and the OS-extended group were found in the following variables: study phase, area, region, number of sites per trial, number of patients per arm, average age, the proportion of males, percentage of squamous cell carcinoma, and smoking history (Table 2).

Exclusion of arms from the OS-reduced group did not improve correlation between OS and PFS. Therefore, further analysis of the OS-reduced group was not conducted.

### Identification of factors influencing OS extended

We selected types of drugs (chemotherapeutic agent, others), study period (1998–2003, 2004–2008), number of patients (<150, ≥150), average age (<63, ≥63), percentage of male (<70, ≥70), percentage of patients with PS1 <60, ≥60), percentage of patients with stage IV disease (<80, ≥80), and percentage of patients with squamous cell carcinoma (<30, ≥30) as potential influencing factors (Supplement Table 1). The number of arms, for which information about region, number of sites, and smoking history was small, was excluded from the logistic regression analyses.

On univariate logistic regression analyses using these potential factors, we identified variables such as number of patients, average age, percentage of males, and histological cancer type as factors potentially influencing extension of OS with statistically significant association (Table 4). On further multivariate logistic regression analyses, number of patients less than 150 per study arm, average age younger than 63 years, and percentage of patients in the study arm with squamous cell carcinoma of <30 % were identified as statistically significant influencing factors for extended OS as shown in Table 4.

**Table 4:** Influencing factors identified by univariate and multiple analysis for the OS-extended group

Characteristics	Category	Univariate analysis			Multivariate analysis		
		OR	95 % CI	*p* value	OR	95 % CI	*p* value
Treatments	Chemo only others	1.444	0.544–3.833	0.461	–	–	–
Study period	1998–2003 2004–2008	1.567	0.541–4.538	0.408	–	–	–
Number of patients	<150 ≥150	0.209	0.057–0.762	0.018	0.045	0.007–0.308	0.002
Average of age (year)	<63, ≥63	0.176	0.038–0.811	0.026	0.087	0.010–0.770	0.028
Percentage of male patients (%)	<70 ≥70	0.198	0.066–0.591	0.004	0.218	0.038–1.237	0.086
Percentage of patients with PS 1 (%)	<60 ≥60	1.486	0.539–4.100	0.444	–	–	–
Percentage of patients with stage IV disease (%)	<80 ≥80	0.431	0.157–1.184	0.103	0.243	0.051–1.166	0.077
Percentage of patients with squamous cell carcinoma (%)	<30 ≥30	0.072	0.009–0.566	0.012	0.074	0.007–0.799	0.032

*OR* odds ratio

## Discussion

New treatment strategies, particularly the introduction of molecular-targeted agents and appropriate patient selection based on histology and/or genotyping, have progressed markedly in recent years, and the OS in advanced NSCLC patients has improved (Ramalingam et al. 2011; Kobayashi and Hagiwara 2013; Kaneda et al. 2013). There are now examples of improvement in PFS without an OS benefit (Lima et al. 2009), and an OS benefit without PFS improvement (Cheema and Burkes 2013). Recent well-developed multiple lines of therapies for advanced NSCLC patients after progression with first-line treatment have been associated with an increase in PPS.

We confirmed that OS was more strongly associated with PPS than with PFS among 140 arms of 65 phase II and III clinical trials for first-line treatment of advanced NSCLC. Our results are similar to those previously reported (Hotta et al. 2011; Hayashi et al. 2012). These strong associations between PPS and OS have also been shown in the first-line treatment of advanced colorectal (Petrelli and Barni 2013) and breast cancers (Saad et al. 2010a, b).

Broglio et al. demonstrated that PPS has an important impact on the association between PFS and OS (Broglio and Berry 2009). When PPS is short, PFS benefit results in a statistically significant OS benefit; however, moderate to longer PPS results in reducing or losing an OS benefit in comparative clinical trials of first-line treatment, even if a significant difference in PFS is observed in randomized trials. If using OS as the primary endpoint, subsequent multiple treatments after the experimental treatment should be considered in clinical trial design (collecting data or defining subsequent treatment options) because PPS may be a potential confounding factor. However, none of the reports we reviewed mentioned these details.

Improving OS remains the gold standard of clinical trials. However, when OS benefit is diluted and masked by longer PPS, OS may not be the most appropriate primary endpoint for assessing the clinical effect for first-line treatment. Although PFS is not a very good surrogate of OS, particularly when PPS is long, PFS should be considered as an attractive endpoint, because it is available earlier than OS, is less influenced than OS by competing causes of death, and is not influenced by PPS. Notably, in advanced breast cancer, there have been few phase III trials where OS was used as the primary endpoint (Verma et al. 2011). It has become increasingly common for PFS or TTP to be used as a primary endpoint in recent phase III randomized trials of first-line treatment for advanced breast cancer (Saad et al. 2010a, b; Saad and Katz 2009) and metastatic colorectal cancer (Saad et al. 2010a). In these cancers, it is well known that subsequent line of therapy plays a major role in determining OS (Saad et al. 2010a, b; Tang et al. 2007; Chirila et al. 2012).

In advanced NSCLC, PFS has not been accepted as a surrogate for OS (Laporte et al. 2013). However, the US Food and Drug Administration has a draft guidance regarding the use of PFS as a clinical endpoint, which is likely to be accepted if the observed magnitude of effect is substantial and robust (Food Drug Administration 2011). The recent progress of multi-line treatment after first-line treatment for advanced NSCLC has provided longer PPS, which resulted in a reduced OS benefit as the primary endpoint, as already seen in advanced breast and colorectal cancers (Cufer et al. 2013), and PFS is a common primary endpoint in current randomized clinical trials. A search in clinical trial databases by Soria et al. (2010) found that more than 150 trials use PFS as the primary endpoint in stage III/IV of NSCLC. Schrimpf et al. (2013) proposes that PFS with the addition of some measures like patient-reported outcomes such as QOL and/or treatment toxicity could cover the clinical benefit in NSCLC studies for individualized therapies with clear patient selection. Mandrekar et al. (2010) reported that PFS or failure-free survival at 12 weeks was a stronger predictor of subsequent patient survival than tumor response and proposed that this be used routinely as an endpoint in phase II trials for advanced NSCLC. This could lead to more accurate assessment of the true efficacy of new drugs for advanced NSCLC.

Our meta-analysis with moderate association between PFS and OS suggests that PFS is not an appropriate surrogate for OS. We conducted subgroup analysis to identify factors associated with the longer OS than that estimated from PFS. Among 65 trials, we identified 20 arms of 14 trials as the OS-extended group, wherein the observed OS was 20 % longer than the estimated OS based on PFS. When these 20 arms were excluded from the analysis, the correlation between OS and PFS was improved. It was noteworthy that there were factors in addition to PPS involved in reducing the association. Univariate analysis identified four variables significantly relevant to OS extension (*p* < 0.05): number of patients (<150/arm), mean age (<63 years), percentage of males (<70 %), and histology of NSCLC (<30 % of squamous cell cancer). Multivariate analysis showed that three of these four variables were statistically correlated with OS-extended group: number of patients less than 150 per arm, mean age younger than 63 years and squamous cell cancer <30 %.

The most widely accepted prognosis determinants are disease stage and PS (Mountain 1997). Male gender, age older than 60 years, non-squamous histology, smoking, and weight loss are known to be prognostic factors (Charloux et al. 1997). Therefore, it is not unexpected that age younger than 63 years and squamous cell carcinoma <30 % were influencing factors of OS extension in our findings. PS is a valid prognostic factor (Belbaraka et al. 2010), but was not identified as such in our study. This might be due to the fact that mainly PS 0 or PS 1 patients were enrolled in most of the clinical trials (PS 2 was only seen in 4.2 % of all patients). The percentage of smokers was excluded from the regression analyses because of small sample size, but this could be expected to have an influence on OS in NSCLC clinical trials. Interestingly, no effect of molecular-targeted agents on OS extension was observed, as no statistical significance was observed in either the treatment regimen or the period in which the trials were conducted. In the OS-extended group, the number of trials in which Asian counties were involved was significantly greater in the OS-extended group than in the OS-association group. However, this factor was not analyzed further due to limited sample size. Participation of Asian countries in recent global clinical trials has been increasing, and an affect of region and race might be considered in future clinical trials.

When PFS is used as the primary endpoint in phase II trial and OS is used in phase III trial, our results suggest factors to be considered in protocol design in order to elucidate the true clinical benefit of experimental drugs for the first-line treatment of advanced NSCLC: (1) increasing the number of sites and number of patients which would improve the association between OS and PFS, (2) adjusting patient baseline characteristics, particularly relevant to prognosis factors both in phase II and phase III trial.

Several technical limitations of our study should be acknowledged. First, our study was not based on individuals, and many complex conditions were involved. Second, there was a limitation in terms of available parameters. Conducting trials in Asia seemed to influence OS extension, but small sample size meant this did not provide statistically significant information. Finally, data on subsequent treatment after progression were not available; such data may be very important when PPS is considered as a potential confounder.

In conclusion, we identified number of patients and well-known prognostic factors including age and histological cancer type as factors influencing longer OS. These factors should be considered for patient eligibility, when PFS is used as a surrogate primary endpoint for OS in randomized clinical trials of first-line treatment for patients with advanced NSCLC.

## Electronic supplementary material

Below is the link to the electronic supplementary material.

Supplementary material 1 (DOCX 16 kb)

Supplementary material 2 (DOCX 47 kb)
