# Supplementary material for: Factors affecting the association between overall survival and progression-free survival in clinical trials of first-line treatment for patients with advanced non-small cell lung cancer
Source: J Cancer Res Clin Oncol. 2014 Feb 22;140(5):839–48. doi: 10.1007/s00432-014-1617-3 (PMC3983956; doi:10.1007/s00432-014-1617-3)
Supplement: Supplementary file 1 — Supplementary material 1 (DOCX 16 kb) [file 432_2014_1617_MOESM1_ESM.docx]

Supplement Table 1. Analysis of chi-square test used in 2 × 2 tables.

| **Characteristics** | **Category** | **p-value** |
| --- | --- | --- |
| Number of patients | <200, >=200 | 0.015 |
|  | <150, >=150 | 0.011 |
|  | <100, >=100 | 0.123 |
| Number of sites | <30, >=30 | 0.028 |
|  | <20, >=20 | <0.001 |
|  | <10, >=10 | 0.858 |
| Average of age (years) | <60, >=60 | 0.009 |
|  | <63, >=63 | 0.014 |
|  | <65, >=65 | 0.059 |
| Percentage of male patients | <60, >=60 | <0.001 |
|  | <70, >=70 | 0.002 |
|  | <80, >=80 | 0.088 |
| Percentage of patients with squamous cell carcinoma | <20, >=20 | <0.001 |
|  | <30, >=30 | 0.002 |
|  | <40, >=40 | 0.086 |
| Percentage of smokers | <70, >=70 | <0.001 |
|  | <80, >=80 | 0.006 |
|  | <90, >=90 | 0.197 |
