# Supplementary material for: Factors affecting the association between overall survival and progression-free survival in clinical trials of first-line treatment for patients with advanced non-small cell lung cancer
Source: J Cancer Res Clin Oncol. 2014 Feb 22;140(5):839–48. doi: 10.1007/s00432-014-1617-3 (PMC3983956; doi:10.1007/s00432-014-1617-3)
Supplement: Supplementary file 2 — Supplementary material 2 (DOCX 47 kb) [file 432_2014_1617_MOESM2_ESM.docx]

**Figure legends**

Supplement Figure 1. Correlation between Overall Survival (OS) and Progression Free Survival (PFS) for 120 arms below 1.2 (OS observed/estimated) for first-line treatment for patients with advanced NSCLC.


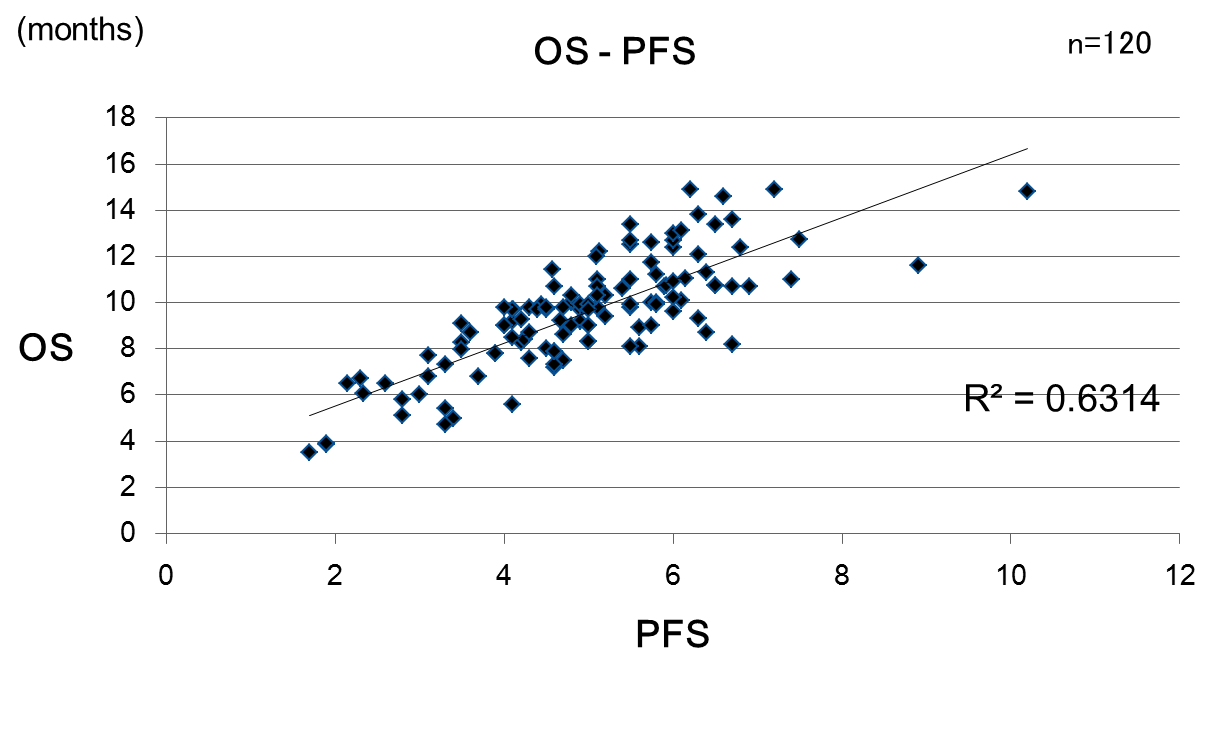


Supplement Figure 1.
